# HLA class I and II gene polymorphisms in Stevens-Johnson syndrome with ocular complications in Japanese

**Published:** 2008-03-17

**Authors:** Mayumi Ueta, Katsushi Tokunaga, Chie Sotozono, Tsutomu Inatomi, Toshio Yabe, Masaki Matsushita, Yoko Mitsuishi, Shigeru Kinoshita

**Affiliations:** 1 Department of Ophthalmology, Kyoto Prefectural University of Medicine, Kyoto, Japan; 2 Department of Human Genetics, Graduate School of Medicine, University of Tokyo, Tokyo, Japan; 3 Tokyo Metropolitan Red Cross Blood Center, Tokyo, Japan; 4 Institute for Biotechnology Research, Wakunaga Pharmaceutical Co., Hiroshima, Japan; 5 Immunogenetic Laboratory, Louis Pasteur Centre for Research, Kyoto, Japan.

## Abstract

**Purpose:**

Stevens-Johnson syndrome (SJS) and toxic epidermal necrolysis (TEN) are acute-onset mucocutaneous diseases induced by infectious agents and/or inciting drugs. Although the pathobiological mechanisms underlying the onset of SJS/TEN have not been fully established, the extreme rarity of cutaneous and ocular surface reactions to drug therapies led us to suspect individual susceptibility. Our previous study of polymorphisms in the HLA-class I genes of 40 Japanese SJS/TEN patients with ocular surface complications showed that in the Japanese, HLA-A*0206 was strongly associated with SJS/TEN. In this study, we investigated the association between HLA class II antigens in addition to HLA class I antigens and SJS/TEN.

**Methods:**

We studied the histocompatibility antigen genes, HLA-A, B, C, DRB1, and DQB1, of 71 Japanese SJS/TEN patients with ocular complications. We also genotyped 113 healthy volunteers for HLA-A, B, C, DRB1, and DQB1. We performed polymerase chain reaction amplification followed by hybridization with sequence-specific oligonucleotide probes (PCR-SSO) using commercial bead-based typing kits.

**Results:**

HLA-A*0206 was strongly associated with SJS/TEN. HLA-A*1101 was inversely associated. HLA-B*5901 exhibited a high odds ratio for SJS/TEN with ocular complications. However, when we corrected the p-value for the number of alleles detected (n=29), the results ceased to be significant. There was no association between HLA-C and SJS/TEN. There was also no significant association between HLA-DRB1 and SJS/TEN. HLA-DQB1*0502 was negatively and weakly associated with SJS/TEN although correction of the p-value for the number of alleles detected rendered the result not significant.

**Conclusions:**

Because our findings are completely different from data reported on Caucasian patients, they suggest strong ethnic differences in the HLA-SJS associations.

## Introduction

Stevens-Johnson syndrome (SJS), an acute inflammatory vesiculobullous reaction of the skin and mucous membranes first described in 1922 [1], is commonly associated with infectious agents and/or inciting drugs [2,3]. In patients with extensive skin detachment and a poor prognosis, the condition is called toxic epidermal necrolysis (TEN) [4]. Although erythema multiforme (EM), SJS, and TEN were formerly accepted as part of a single “EM spectrum,” a retrospective analysis of the type and distribution of skin lesions and the extent of epidermal detachment identified EM major and SJS/TEN as two separate clinical entities that differed with respect to histopathologic changes and etiology [5]. The annual incidence of SJS and TEN has been estimated as 0.4–1 and 1–6 cases per million persons, respectively [3,6]; the mortality rate is 3% and 27%, respectively [7]. Although rare, these reactions carry high morbidity and mortality rates and often result in severe and definitive sequelae such as vision loss. The pathobiological mechanisms underlying the onset of SJS/TEN have not been fully established although the involvement of immune mechanisms [8,9], especially altered drug metabolism [10] and infections such as *Mycoplasma pneumoniae* [11], has been suggested. The extreme rarity of cutaneous and ocular surface reactions to drug therapies led us to suspect individual susceptibility.

In the acute stage, SJS/TEN patients manifest severe conjunctivitis and persistent corneal epithelial defects due to ocular surface inflammation with vesiculobullous skin lesions. In the chronic stage, ocular surface complications, such as conjunctival invasion into the cornea due to corneal epithelial stem cell deficiency, symblepharon, ankyloblepharon, and in some instances, keratinization of the ocular surface, persist despite the healing of the skin lesions [12]. Moreover, we observed that more than 95% of patients with SJS/TEN with ocular complications had lost their fingernails in the acute or sub-acute stage and that some continue to have transformed nails even after the healing of their skin lesions [2,13]. SJS/TEN is one of the most devastating ocular surface diseases, leading to corneal damage and loss of vision. The reported incidence of ocular complications in SJS/TEN is 50%–68% [3,7]. In this study, we focused on patients with SJS/TEN accompanied by ocular surface complications.

**Table 1:** HLA-A alleles and SJS/TEN with ocular complications.

**HLA-A alleles**	**Carrier frequency**				**Allele frequency**			
	**SJS (n=71)**	**Normal (n=113)**	**p-value (Χ^2^)**	**Odds ratio**	**SJS (n=142)**	**Normal (n=226)**	**p-value (Χ^2^)**	**Odds ratio**
*0101	0	0.035	0.16	-	0	0.018	0.3	-
	(0/71)	(4/113)			(0/142)	(4/226)		
*0201	0.268	0.221	0.47	-	0.155	0.111	0.22	-
	(19/71)	(25/113)			(22/142)	(25/226)		
*0206	0.423	0.15	0.00004	4.1	0.225	0.084	0.0001	3.2
	(30/71)	(17/113)	*		(32/142)	(19/226)	*	
*0207	0.07	0.08	1	-	0.035	0.04	1	-
	(5/71)	(9/113)			(5/142)	(9/226)		
*0301	0.014	0	0.39	-	0.007	0	0.39	-
	(1/71)	(0/113)			(1/142)	(0/226)		
*1101	0.056	0.204	0.005	0.23	0.028	0.115	0.003	0.22
	(4/71)	(23/113)			(4/142)	(26/226)	*	
*2402	0.521	0.566	0.55	-	0.296	0.332	0.47	-
	(37/71)	(64/113)			(42/142)	(75/226)		
*2601	0.099	0.115	0.81	-	0.049	0.062	0.65	-
	(7/71)	(13/113)			(7/142)	(14/226)		
*2602	0.056	0.035	0.49	-	0.028	0.018	0.49	-
	(4/71)	(4/113)			(4/142)	(4/226)		
*2603	0.014	0.027	1	-	0.007	0.013	1	-
	(1/71)	(3/113)			(1/142)	(3/226)		
*3001	0.014	0.009	1	-	0.007	0.004	1	-
	(1/71)	(1/113)			(1/142)	(1/226)		
*3101	0.099	0.186	0.14	-	0.049	0.093	0.16	-
	(7/71)	(21/113)			(7/142)	(21/226)		
*3303	0.225	0.212	0.84	-	0.113	0.111	0.95	-
	(16/71)	(24/113)			(16/142)	(25/226)		

HLA-A*0206 was strongly associated with SJS/TEN with ocular complications (carrier frequency: p<0.00005, corrected p value (Pc)<0.0005, OR=4.1; allele frequency: p<0.0005, Pc<0.005, OR=3.2). HLA-A*1101 was inversely associated (carrier frequency: p<0.01, Pc=0.07, OR=0.23; allele frequency: p<0.005, Pc<0.05, OR=0.22). SJS/TEN patients in this study consisted of 40 previously analyzed patients and 31 new patients. These results validate the strong association between HLA-A*0206 and SJS/TEN that we reported previously [13].

**Table 2:** HLA-B alleles and SJS/TEN with ocular complications.

**HLA-B alleles**	**Carrier frequency**				**Allele frequency**			
	**SJS (n=71)**	**Normal (n=113)**	**p**	**Odds ratio**	**SJS (n=142)**	**Normal (n=226)**	**p**	**Odds ratio**
*0702	0.113	0.106	1	-	0.056	0.053	1	-
	(8/71)	(12/113)			(8/142)	(12/226)		
*1301	0.07	0.027	0.26	-	0.035	0.013	0.27	-
	(5/71)	(3/113)			(5/142)	(3/226)		
*1302	0	0.009	1	-	0	0.004	1	-
	(0/71)	(1/113)			(0/142)	(1/226)		
*1501	0.042	0.115	0.11	-	0.028	0.058	0.21	-
	(3/71)	(13/113)			(4/142)	(13/226)		
*1511	0	0.009	1	-	0	0.004	1	-
	(0/71)	(1/113)			(0/142)	(1/226)		
*1518	0.014	0.035	0.65	-	0.007	0.018	0.65	-
	(1/71)	(4/113)			(1/142)	(4/226)		
*1527	0.014	0	0.39	-	0.007	0	0.39	-
	(1/71)	(0/113)			(1/142)	(0/226)		
*2704	0.014	0	0.39	-	0.007	0	0.39	-
	(1/71)	(0/113)			(1/142)	(0/226)		
*3501	0.211	0.15	0.29	-	0.106	0.084	0.49	-
	(15/71)	(17/113)			(15/142)	(19/226)		
*3701	0	0.027	0.29	-	0	0.013	0.29	-
	(0/71)	(3/113)			(0/142)	(3/226)		
*3802	0	0.009	1	-	0	0.004	1	-
	(0/71)	(1/113)			(0/142)	(1/226)		
*3901	0.085	0.071	0.78	-	0.042	0.035	0.78	-
	(6/71)	(8/113)			(6/142)	(8/226)		
*3902	0.014	0.018	1	-	0.007	0.009	1	-
	(1/71)	(2/113)			(1/142)	(2/226)		
*4001	0.169	0.124	0.39	-	0.092	0.062	0.29	-
	(12/71)	(14/113)			(13/142)	(14/226)		
*4002	0.127	0.133	1	-	0.07	0.066	0.88	-
	(9/71)	(15/113)			(10/142)	(15/226)		
*4003	0.014	0	0.39	-	0.007	0	0.39	-
	(1/71)	(0/113)			(1/142)	(0/226)		
*4006	0.099	0.097	1	-	0.049	0.049	1	-
	(7/71)	(11/113)			(7/142)	(11/226)		
*4402	0.014	0	0.39	-	0.007	0	0.39	-
	(1/71)	(0/113)			(1/142)	(0/226)		
*4403	0.225	0.204	0.72	-	0.12	0.106	0.69	-
	(16/71)	(23/113)			(17/142)	(24/226)		
*4601	0.085	0.115	0.62	-	0.042	0.062	0.49	-
	(6/71)	(13/113)			(6/142)	(14/226)		
*4801	0.028	0.088	0.13	-	0.014	0.044	0.14	-
	(2/71)	(10/113)			(2/142)	(10/226)		
*5101	0.197	0.124	0.18	-	0.113	0.066	0.12	-
	(14/71)	(14/113)			(16/142)	(15/226)		
*5102	0.028	0.009	0.56	-	0.014	0.004	0.56	-
	(2/71)	(1/113)			(2/142)	(1/226)		
*5201	0.127	0.212	0.17	-	0.07	0.115	0.16	-
	(9/71)	(24/113)			(10/142)	(26/226)		
*5401	0.042	0.133	0.07	-	0.021	0.066	0.08	-
	(3/71)	(15/113)			(3/142)	(15/226)		
*5502	0.028	0.044	0.71	-	0.014	0.022	0.71	-
	(2/71)	(5/113)			(2/142)	(5/226)		
*5601	0.028	0.027	1	-	0.014	0.013	1	-
	(2/71)	(3/113)			(2/142)	(3/226)		
*5901	0.113	0.018	0.01	7	0.056	0.009	0.02	6.7
	(8/71)	(2/113)			(8/142)	(2/226)		
*6701	0	0.035	0.16	-	0	0.018	0.3	-
	(0/71)	(4/113)			(0/142)	(4/226)		

HLA-B*5901 exhibited a high odds ratio for SJS/TEN with ocular complications (carrier frequency: p<0.05, Pc=0.42, OR=7.0; allele frequency: p<0.05, Pc=0.46, OR=6.7). However, when we corrected the p-value for the number of alleles detected (n=29), the results ceased to be significant.

**Table 3:** HLA-C alleles and SJS/TEN with ocular complications.

**HLA-C alleles**	**Carrier frequency**				**Allele frequency**			
	**SJS (n=71)**	**Normal (n=113)**	**p**	**Odds ratio**	**SJS (n=142)**	**Normal (n=226)**	**p**	**Odds ratio**
*0102	0.268	0.327	0.4	-	0.141	0.168	0.48	-
	(19/71)	(37/113)			(20/142)	(38/226)		
*0303	0.239	0.168	0.24	-	0.12	0.093	0.41	-
	(17/71)	(19/113)			(17/142)	(21/226)		
*0304	0.366	0.23	0.046	1.9	0.197	0.124	0.057	-
	(26/71)	(26/113)			(28/142)	(28/226)		
*0401	0.07	0.115	0.45	-	0.035	0.058	0.46	-
	(5/71)	(13/113)			(5/142)	(13/226)		
*0501	0.014	0	0.39	-	0.007	0	0.39	-
	(1/71)	(0/113)			(1/142)	(0/226)		
*0602	0	0.035	0.16	-	0	0.018	0.3	-
	(0/71)	(4/113)			(0/142)	(4/226)		
*0702	0.211	0.212	0.99	-	0.12	0.119	0.99	-
	(15/71)	(24/113)			(17/142)	(27/226)		
*0704	0.014	0.018	1	-	0.007	0.009	1	-
	(1/71)	(2/113)			(1/142)	(2/226)		
*0801	0.099	0.168	0.28	-	0.049	0.088	0.22	-
	(7/71)	(19/113)			(7/142)	(20/226)		
*0803	0.028	0.053	0.71	-	0.014	0.027	0.71	-
	(2/71)	(6/113)			(2/142)	(6/226)		
*1202	0.127	0.212	0.17	-	0.07	0.115	0.16	-
	(9/71)	(24/113)			(10/142)	(26/226)		
*1402	0.141	0.097	0.37	-	0.078	0.053	0.35	-
	(10/71)	(11/113)			(11/142)	(12/226)		
*1403	0.225	0.204	0.72	-	0.113	0.106	0.85	-
	(16/71)	(23/113)			(16/142)	(24/226)		
*1502	0.099	0.044	0.22	-	0.049	0.022	0.23	-
	(7/71)	(5/113)			(7/142)	(5/226)		

There was no association between HLA-C and SJS/TEN with ocular complications.

Our previous study of polymorphisms in the HLA-class I genes (HLA-A, B, C) of 40 Japanese SJS/TEN patients with ocular surface complications showed that in the Japanese, HLA-A*0206 was strongly associated with SJS/TEN with ocular surface complications [13]. We also documented that in Japanese SJS/TEN patients with ocular surface complications there was an association with TLR3 polymorphisms [2] and with IL4R polymorphisms [14]. Thus, genetic factors play an important role in an integrated etiology of SJS/TEN. However, HLA class II gene polymorphisms of Japanese SJS/TEN patients have not yet been reported.

Under the hypothesis of an immunologic reaction in susceptible individuals, we studied HLA class II (DRB1 and DQB1) gene polymorphisms in addition to HLA class I (HLA-A, B, C) in 71 Japanese SJS/TEN patients with ocular surface complications.

## Methods

### Patients

This study was approved by the Institutional Review Board of Kyoto Prefectural University of Medicine, Kyoto, Japan. All experimental procedures were conducted in accordance with the principles set forth in the Helsinki Declaration. The purpose of the research and the experimental protocols were explained to all participants, and their written informed consent was obtained.

For HLA genotyping, we enrolled 71 Japanese patients with SJS/TEN in the chronic or sub-acute phase; all presented with ocular surface complications. The diagnosis of SJS/TEN was based on a confirmed history of acute-onset high fever, serious mucocutaneous illness with skin eruptions, and involvement of at least two mucosal sites including the ocular surface. The average patient age was 45.8 ± 17.4 years; the male:female ratio was 31:40.

### Controls

The normal control group consisted of 113 healthy volunteer blood donors for HLA class I (A, B, C) and HLA class II (DRB1, DQB1) for genotyping. All volunteers were Japanese residing in Japan.

### HLA genotyping

We studied the histocompatibility antigen genes HLA-A, B, C, DRB1, and DQB1 of 71 Japanese SJS/TEN patients with ocular complications. We also genotyped 113 healthy volunteers for HLA-A, B, C, DRB1, and DQB1. These alleles were detected by the polymerase chain reaction (PCR)-Luminex typing method using the WAKFlow HLA typing kit (Wakunaga, Hiroshima, Japan). First, the target DNA was amplified by polymerase chain reactions with biotinylated primers specifically designed for each HLA locus. Then, the PCR product was denatured and hybridized to complementary oligonucleotide probes immobilized on fluorescent coded microsphere beads. At the same time, biotinylated PCR product was labeled with phycoerythrin-conjugated streptavidin and immediately examined with the Luminex 100 system. Genotype determination and data analysis were performed automatically, using the WAKFlow typing software.

**Table 4:** HLA-DRB1 alleles and SJS/TEN with ocular complications.

**HLA-DRB1 alleles**	**Carrier frequency**				**Allele frequency**			
	**SJS (n=71)**	**Normal (n=113)**	**p**	**Odds ratio**	**SJS (n=142)**	**Normal (n=226)**	**p**	**Odds ratio**
*0101	0.113	0.097	0.81	-	0.056	0.049	0.81	-
	(8/71)	(11/113)			(8/142)	(11/226)		
*0401	0.028	0.018	0.64	-	0.014	0.009	0.64	-
	(2/71)	(2/113)			(2/142)	(2/226)		
*0403	0.028	0.053	0.71	-	0.014	0.031	0.49	-
	(2/71)	(6/113)			(2/142)	(7/226)		
*0404	0.014	0.009	1	-	0.007	0.004	1	-
	(1/71)	(1/113)			(1/142)	(1/226)		
*0405	0.197	0.248	0.43	-	0.106	0.131	0.44	-
	(14/71)	(28/113)			(15/142)	(30/226)		
*0406	0.028	0.062	0.49	-	0.014	0.031	0.49	-
	(2/71)	(7/113)			(2/142)	(7/226)		
*0407	0	0.035	0.16	-	0	0.018	0.3	-
	(0/71)	(4/113)			(0/142)	(4/226)		
*0410	0.028	0.018	0.64	-	0.014	0.009	0.64	-
	(2/71)	(2/113)			(2/142)	(2/226)		
*0701	0.014	0.009	1	-	0.007	0.004	1	-
	(1/71)	(1/113)			(1/142)	(1/226)		
*0802	0.113	0.062	0.27	-	0.056	0.031	0.28	-
	(8/71)	(7/113)			(8/142)	(7/226)		
*0803	0.239	0.133	0.06	-	0.12	0.071	0.11	-
	(17/71)	(15/113)			(17/142)	(16/226)		
*0901	0.301	0.31	1	-	0.176	0.168	0.84	-
	(22/71)	(35/113)			(25/142)	(38/226)		
*1001	0	0.009	1	-	0	0.004	1	-
	(0/71)	(1/113)			(0/142)	(1/226)		
*1101	0.07	0.027	0.26	-	0.035	0.013	0.27	-
	(5/71)	(3/113)			(5/142)	(3/226)		
*1201	0.028	0.062	0.49	-	0.014	0.031	0.49	-
	(2/71)	(7/113)			(2/142)	(7/226)		
*1202	0.085	0.035	0.19	-	0.042	0.018	0.19	-
	(6/71)	(4/113)			(6/142)	(4/226)		
*1301	0.014	0.009	1	-	0.007	0.004	1	-
	(1/71)	(1/113)			(1/142)	(1/226)		
*1302	0.169	0.195	0.66	-	0.085	0.097	0.68	-
	(12/71)	(22/113)			(12/142)	(22/226)		
*1401	0.028	0.053	0.71	-	0.014	0.027	0.72	-
	(2/71)	(6/113)			(2/142)	(6/226)		
*1403	0.042	0.062	0.74	-	0.021	0.031	0.75	-
	(3/71)	(7/113)			(3/142)	(7/226)		
*1405	0.056	0.062	1	-	0.028	0.031	1	-
	(4/71)	(7/113)			(4/142)	(7/226)		
*1406	0.014	0.018	1	-	0.007	0.009	1	-
	(1/71)	(2/113)			(1/142)	(2/226)		
*1501	0.183	0.106	0.14	-	0.092	0.058	0.21	-
	(13/71)	(12/113)			(13/142)	(13/226)		
*1502	0.127	0.186	0.31	-	0.07	0.102	0.31	-
	(9/71)	(21/113)			(10/142)	(23/226)		
*1602	0	0.035	0.16	-	0	0.018	0.3	-
	(0/71)	(4/113)			(0/142)	(4/226)		

There was no significant association between HLA-DRB1 and SJS/TEN with ocular complications.

### Statistical methods

For statistical analysis to compare carrier frequency and gene frequency, we used the χ^2^-test for statistical analysis when the sample number was 10 and more than 10 and used the Fischer’s exact test when the sample number was less than 10. The odds ratio (OR) with 95% confidence intervals (95% CI) was calculated using Labo Server software (World Fusion, Tokyo, Japan). Each allele was assessed as an independent variable and separate p values were calculated. A p value of <0.05 was regarded as significant. In addition, the p values were corrected for the number of alleles tested.

**Table 5:** HLA-DQB1 alleles and SJS/TEN with ocular complications.

**HLA-DQB1 alleles**	**Carrier frequency**				**Allele frequency**			
	**SJS (n=71)**	**Normal (n=113)**	**p**	**Odds ratio**	**SJS (n=142)**	**Normal (n=226)**	**p**	**Odds ratio**
*0201	0.014	0.009	1	-	0.007	0.004	1	-
	(1/71)	(1/113)			(1/142)	(1/226)		
*0301	0.254	0.212	0.52	-	0.141	0.106	0.32	-
	(18/71)	(24/113)			(20/142)	(24/226)		
*0302	0.169	0.177	0.89	-	0.085	0.093	0.78	-
	(12/71)	(20/113)			(12/142)	(21/226)		
*0303	0.296	0.354	0.41	-	0.169	0.195	0.54	-
	(21/71)	(40/113)			(24/142)	(44/226)		
*0401	0.197	0.239	0.51	-	0.106	0.128	0.51	-
	(14/71)	(27/113)			(15/142)	(29/226)		
*0402	0.056	0.053	1	-	0.028	0.027	1	-
	(4/71)	(6/113)			(4/142)	(6/226)		
*0501	0.113	0.097	0.81	-	0.056	0.053	1	-
	(8/71)	(11/113)			(8/142)	(12/226)		
*0502	0	0.08	0.01	0	0	0.04	0.01	0
	(0/71)	(9/113)			(0/142)	(9/226)		
*0503	0.085	0.062	0.57	-	0.042	0.031	0.57	-
	(6/71)	(7/113)			(6/142)	(7/226)		
*0601	0.352	0.274	0.26	-	0.19	0.164	0.63	-
	(25/71)	(31/113)			(26/142)	(37/226)		
*0602	0.155	0.106	0.33	-	0.078	0.058	0.45	-
	(11/71)	(12/113)			(11/142)	(13/226)		
*0603	0.014	0.009	1	-	0.007	0.004	1	-
	(1/71)	(1/113)			(1/142)	(1/226)		
*0604	0.169	0.195	0.66	-	0.085	0.097	0.68	-
	(12/71)	(22/113)			(12/142)	(22/226)		

HLA-DQB1*0502 showed a tendency of negative association with SJS/TEN with ocular complications (carrier frequency: p<0.05, Pc=0.17, OR=0; allele frequency: p<0.05, Pc=0.19, OR=0). Although none of the 71 SJS/TEN patients and 10 of the 117 healthy volunteers (8.5%) had the HLA-DQB1*0502 allele, the correction of the p-value for the number of alleles detected (n=14) rendered the result not significant.

## Results

As shown in Table 1, HLA-A*0206 was strongly associated with SJS/TEN with ocular complications (carrier frequency: p<0.00005, corrected p value (Pc)<0.0005, OR=4.1; allele frequency: p<0.0005, Pc<0.005, OR=3.2). HLA-A*1101 was inversely associated (carrier frequency: p<0.01, Pc=0.07, OR=0.23; allele frequency: p<0.005, Pc<0.05, OR=0.22). SJS/TEN patients in this study consisted of 40 previously analyzed patients and 31 new patients. These results validate the strong association between HLA-A*0206 and SJS/TEN that we reported previously [13].

Table 2 shows the results on HLA-B alleles. HLA-B*5901 exhibited a high odds ratio for SJS/TEN with ocular complications (carrier frequency: p<0.05, Pc=0.42, OR=7.0; allele frequency: p<0.05, Pc=0.46, OR=6.7). However, when we corrected the p-value for the number of alleles detected (n=29), the results ceased to be significant.

There was no association between HLA-C and SJS/TEN with ocular complications (Table 3). There was also no significant association between HLA-DRB1 and SJS/TEN with ocular complications (Table 4).

HLA-DQB1*0502 showed a tendency of negative association with SJS/TEN with ocular complications (carrier frequency: p<0.05, Pc=0.17, OR=0; allele frequency: p<0.05, Pc=0.19, OR=0); Table 5). Although none of the 71 SJS/TEN patients and 10 of the 117 healthy volunteers (8.5%) had the HLA-DQB1*0502 allele, the correction of the p-value for the number of alleles detected (n=14) rendered the result not significant.

We used the χ^2^-test for statistical analysis when the sample number was 10 and more than 10 and used the Fischer’s exact test when the sample number was less than 10. Carrier frequency is “frequency of the person with the allele at the population level,” and allele frequency is “frequency of alleles at the population level” [15].

## Discussion

Analysis of our 71 Japanese patients showed that HLA-A*0206 was strongly associated with SJS/TEN with ocular complications. On the other hand, HLA-A*1101 was negatively associated. We postulate that the decreased B*5401 frequency in the patients is attributable to its linkage disequilibrium with A*1101. We also found that HLA-B*5901 was weakly associated with SJS/TEN with ocular complications although when we corrected the p-value for the number of alleles detected, the result ceased to be significant. We postulate that B*5901 could be a risk factor independent of A*0206 because only one patient had both alleles. Interestingly, none of the 71 SJS/TEN patients but 10 of the 117 volunteers (8.5%) had the HLA-DQB1*0502 allele. HLA-DQB1*0502 was also weakly associated with SJS/TEN with ocular complications although correction of the p-value for the number of alleles detected rendered the result not significant.

Regarding HLA-class I, previous reports from the United States [16] and France [17] showed that the HLA-B12 (HLA-Bw44) antigen was significantly increased in Caucasian SJS patients. In our study population, we did not find an association with HLA-B12 probably because in Caucasians, the HLA-B12 antigen is primarily coded by HLA-B*4402 whereas in the Japanese, it is almost exclusively coded by HLA-B*4403 [15]. HLA-A*0206, strongly associated with SJS/TEN with ocular complications in the Japanese, is absent in Caucasians. While we were unable to identify the causative drug(s) unequivocally, we suspect that antibiotics, cold remedies, or non-steroid anti-inflammatory drugs were involved in some of our patients. Limiting carbamazepine-induced SJS, the HLA-B*1502 allele was documented to show a very strong association [18].

With respect to HLA-class II, Power et al. [19] reported that HLA-DQB1*0601 was associated with Caucasian patients with ocular complications of SJS. In French SJS/TEN patients, HLA-DR antigens (DR) was not associated at all [16]. Different from the findings of others, we found that in our Japanese patients, there is no significant association between SJS/TEN and HLA-DQB1*0601.

Thus, our findings suggest strong ethnic differences in the association of SJS/TEN with HLA (Table 6). Because SJS/TEN is a rare condition probably with a complex genetic inheritance background, specific combinations of genes and certain environmental factors may be required for the manifestation of this rare phenotype. Since the strong association of specific HLA antigens with SJS with ocular complications may be a clue to understanding its basic pathobiology, we are attempting to develop a reliable test for identifying individuals susceptible to SJS with ocular complications.

**Table 6:** Ethnic differences in the association of SJS/TEN with HLA.

**Allele**	**Japanese**		**Caucasian [**19**]**		**Taiwanese [**18**]**	
	**SJS**	**Control**	**SJS**	**Control**	**Carbamazepine induced SJS**	**Control**
A*0206	0.423	0.15	-	(0 ~1.4%)	-	(3.1 ~24%)
B*1502	0	0		(0 ~0.2%)	1	0.086
B*4402	0.014	0	-	(6.7 ~26.5%)	-	0
B*4403	0.225	0.204	-	(6.6 ~20.0%)	-	(0 ~2%)
DQB1*0601	0.352	0.274	0.17	0.03	-	-

Carrier frequency of SJS-associated alleles in Japanese and Caucasian. Data in parentheses are from “Allele Frequency in Worldwide Populations.”
